# The Incidence of First Venous Thromboembolism in and around Pregnancy Using Linked Primary and Secondary Care Data: A Population Based Cohort Study from England and Comparative Meta-Analysis

**DOI:** 10.1371/journal.pone.0070310

**Published:** 2013-07-29

**Authors:** Alyshah Abdul Sultan, Laila J. Tata, Matthew J. Grainge, Joe West

**Affiliations:** Division of Epidemiology and Public Health, University of Nottingham, Nottingham, United Kingdom; Wayne State University School of Medicine, United States of America

## Abstract

**Background:**

Recent linkage between primary and secondary care data has provided valuable information for studying heath outcomes that may initially present in different health care settings. The aim of this study was therefore, twofold: to use linked primary and secondary care data to determine an optimum definition for estimating the incidence of first VTE in and around pregnancy; and secondly to conduct a systematic literature review of studies on perinatal VTE incidence with the purpose of comparing our estimates.

**Methods:**

We used primary care data from the Clinical Practice Research Datalink (CPRD), which incorporates linkages to secondary care contained within Hospital Episode Statistics (HES) between 1997 and 2010 to estimate the incidence rate of VTE in the antepartum and postpartum period. We systematically searched the literature on the incidence of VTE during antepartum and postpartum periods and performed a meta-analysis to provide comparison.

**Findings:**

Using combined CPRD and HES data and a restrictive VTE definition, the absolute rate during the antepartum period and first six weeks postpartum (early postpartum) were 99 (95%CI 85–116) and 468 (95%CI 391–561) per 100,000 person-years respectively. These were comparable to the pooled estimates from our meta-analysis (using studies after 2005) during the antepartum period (118/100,000 person-years) and early postpartum (424/100,000 person-years). When we used only secondary care data to identify VTE events, incidence was lower during the early postpartum period (308/100,000 person-years), whereas relying only on primary care data lead to lower incidence during the time around delivery, but higher rates during the postpartum period (558/100,000 person-years).

**Conclusion:**

Using combined CPRD and HES data gives estimates of the risk of VTE in and around pregnancy that are comparable to the existing literature. It also provides more accurate estimation of the date of VTE diagnosis which will allow risk stratification during specific pregnancy and postpartum periods.

## Introduction

Venous thromboembolism (VTE) is a serious complication of pregnancy, however due to the low incidence of pregnancy related VTEs and uncertainties over risk factors, prospective studies are unlikely to be done because of prohibitive costs. Therefore, to study its occurrence and risk factors, studies using routine data have been used as they provide sufficient power and population coverage to give robust and generalisable estimates [1], [2]. The recent linkage of electronic primary data in the Clinical Practice Research Datalink (CPRD) with the secondary care English Hospital Episode Statistics database (HES) may be useful in providing valuable information on maternal risk factors for VTE including hospitalisation, life style related factors and co-morbidities. However, there have been no studies done using these linked data to quantify the incidence of VTE in and around pregnancy. Furthermore no studies have assessed the disparities between the results from secondary and primary care data in a standalone fashion versus when used together. Additionally, defining VTE may be a concern as currently there have been no studies done to validate VTE in linked primary and secondary data. Whilst VTE has been previously validated in primary care data in non-pregnant women with a positive predictive value (PPV) of 84% [3], it is important to assess whether secondary care data add any further information on VTE events around pregnancy especially in the United Kingdom (UK) where almost all women deliver in hospital. For instance, the previous validation study does not give an indication of the negative predictive value and we cannot ignore the potential for the incidence of VTE recorded in primary care to be underestimated if certain VTE cases are solely recorded in secondary care. Further validation is also required to assess potential false positive diagnoses in the peripartum period.

One way of assessing clinical information captured on VTE during antepartum and postpartum periods is to compare estimates of incidence to previous studies of VTE incidence. However to-date, there has not been any formal data synthesis of these previous studies nor an attempt at providing pooled estimates of the incidence of VTE during the antepartum and postpartum period. The aim of this study was therefore, twofold: to use linked primary and secondary care data to determine an optimum definition for estimating the incidence of first VTE in and around pregnancy; and secondly to conduct a systematic literature review of studies on perinatal VTE incidence with the purpose of comparison to our estimates.

## Methods

### Databases and study population

#### Hospital Episode Statistics (HES)

The Hospital episode statistics dataset (HES) [4] contains details of all hospital admissions to National Health Service (NHS) hospitals in England. It contains demographic data along with information on discharge diagnoses and procedures which are coded using International Classification of Diseases (ICD) version 10 and Operation and Procedure Coding Supplement (OPCS) version 4 respectively. All diagnoses within the hospitalisation period (i.e. time they are admitted until the time they are discharged from hospital) are recorded within episodes (time period during which a patient is under a particular consultant). We also used HES maternity data and contains data on births across England and is the primary source of maternity statistics in England which have been validated [5].

#### Clinical Practice Research Datalink

The Clinical Practice Research Datalink (CPRD) [6] is a computerized primary health care database containing demographic, medical, prescription and lifestyle related information from anonymised patient records across the UK. The data are subjected to quality checks and only the data which is of high quality is used for research. The GPRD has been extensively validated for a wide range of chronic diagnoses and consistently found to be accurate [7], [8].

#### Linkage

The anonymised patient identifiers from CPRD and HES have been linked by a trusted third party using NHS number, date of birth and gender [9]. As HES only covers English hospitals, practices from Northern Island, Wales and Scotland were excluded. The primary care CPRD does not use a set sampling approach to for its linkage with secondary care HES data. It is based on practices that have consented to be linked to HES in CPRD. However all patients within a consented practice are included. Additionally our comparison of CPRD-HES linked data to Office for National Statistics (ONS) data showing the age distribution of the UK population has demonstrated similarities [10]. For this study, we used 51% of the CPRD practices that had linked HES data for their registered patients. We identified women of reproductive age (15–44 years old) between 1997 and 2010 registered within the CPRD-HES linked practices as this was the time for which HES linked data were available.

### Defining venous thromboembolism

VTE diagnosis codes (including pulmonary embolism (PE) or deep vein thrombosis (DVT)) were extracted from women's primary care data using medical Read codes. From HES, all women with an ICD-10 code of venous thromboembolism including pulmonary embolism (I26.0, I26.9), deep vein thrombosis (I80.1–I80.9) and portal vein thrombosis (I82.0–I82.9) were extracted. ICD-10 codes of VTE specifically related to pregnancy or postpartum (O22.2, O22.3, O87.1, O87.0, O08.2 and O88.2) were also extracted. Information from either or both primary and secondary care data sources was used to define a VTE event and the first VTE diagnosis recorded in either data source was considered as the incident date. We assessed only the first recorded VTE during the study period and all subsequent VTEs were excluded. We then developed the following three VTE definitions;

#### Definition A

Our most stringent definition included only VTE diagnoses supported by prescription or evidence of anticoagulant therapy (with either warfarin or unfractionated heparin or low molecular weight heparin) within 90 days of the event or death within 30 days of the event. Given the restricted use of oral anticoagulants (e.g. warfarin) during the antepartum period due to its teratogenicity, the majority of cases during the antepartum period were confirmed based on heparin prescriptions.

#### Definition B

This consisted cases where signs or symptoms of DVT (e.g. leg pain, calf pain), PE (e.g. chest pain, shortness of breath) or diagnostic tests for VTE (e.g. d-dimer, Ventilation-Perfusion (VQ) scan, Computed Tomography (CT)-scan, venography) had been recorded between 15 days before and 15 days after a first recorded diagnosis of VTE, but there was no evidence of anticoagulant therapy. Cases were also included if they had VTE diagnoses in both primary and secondary care up to 60 days apart, a cut-off based on the initial examination of the recording of VTE in both datasets and prior work we and others have published on identifying acute medical events in linked data [11], [12].

#### Definition C

This included all other diagnoses of VTE that did not fit the criteria for VTE definitions A or B. Specifically, all VTE diagnoses with no accompanying anticoagulant prescription, medical code indicating anticoagulant therapy, death, signs or symptoms of VTE, diagnostic tests for VTE. These cases were only recorded in one data source (HES or CPRD).

### Defining pregnancy and associated time periods

Information on birth outcome (including live birth and stillbirth) was identified using the mother's delivery records in HES maternity data. For each pregnancy resulting in live or stillbirth, the date of delivery was extracted from the OPCS version 4 codes for delivery (e.g. emergency caesarean section, spontaneous vaginal delivery). The date of conception was defined by subtracting the length of gestation from the date of delivery. For those with no information on length of gestation (33%), 40 weeks was assigned. This corresponds to that of the majority of pregnant women in the National Health Service's (NHS) maternity statistics for England [13]. Women's follow-up time between age 15–44 years was divided into time associated with pregnancy (defined from the date of any conceptions she had during follow-up until 12 weeks postpartum) and “non-pregnant periods” (all other available follow-up time, which included all time for women who were never pregnant during the study period as previously described [14]). If the VTE event was recorded during the same hospital admission as the women's delivery (which accounted for 11% of all VTE events), there was thus potential for misclassifying the timing in relation to delivery. As 91% of deliveries occurred on the day women were admitted to hospital for delivery or on the day after and the median duration of hospital stay for delivery was only 2 days, the time associated with pregnancy was divided into the antepartum period (trimesters from the date of conception until 2 days before the date of delivery), time around delivery (1 day before until 2 days after delivery) and the postpartum period which was defined from 3 days after delivery until 12 weeks postpartum. The postpartum period was subdivided into individual weeks and also into early (first six weeks) and late (second six weeks) postpartum) period.

### Statistical analyses

#### Cohort analysis in linked data

The absolute rates (AR) of VTE per 100,000 person-years and 95% Confidence Interval (CI) were calculated for the antepartum period, time around delivery, postpartum period and non-pregnant periods using VTE definitions A, B and C separately. This was done by dividing the total number of VTE events by person-years of follow-up. We then restricted the analysis to first VTEs identified only in primary care data and then VTEs only in secondary care data to compare these with the overall estimates using both sources.

#### Systematic review and meta-analysis of existing VTE incidence studies

For the purpose of comparing our rates to the existing literature, we systematically reviewed previous studies that have estimated VTE incidence among pregnant women or during the postpartum period. We searched MEDLINE and Embase for studies published between January 1960 and January 2013, combining a similar search strategy to that used in our previously published VTE systematic review [15] with an adapted version of the Cochrane Pregnancy Group search strategy to obtain pregnancy studies [16]. The strategy used for MEDLINE and Embase is summarised in Table S1 and S2 respectively. We included studies only if they had estimated the rate of VTE in pregnant and postpartum women in a manner that allowed us to extract the data for the purposes of meta-analysis. Studies' abstracts were independently reviewed for selection by two investigators (AAS and JW) with differences resolved by consensus.

For each study included in our meta-analysis, the natural logarithm of the incidence rate of VTE per 100,000 person-years was obtained along with the standard errors (1/√VTE events). For studies reporting rates of VTE per 100,000 pregnancies we converted this into person-years of antepartum time by multiplying the number of pregnancies by 0.75. For the meta-analysis, we only assessed the first six weeks after childbirth during the postpartum (although this was only the early postpartum period in our cohort study) as this was the definition of postpartum used in the majority of the included publications. These were then pooled separately for antepartum and postpartum periods assuming random effects using the generic inverse variance method. This method considers the inverse variance of the effect estimates i.e. 1/(standard error)^2^ as the weight given to each study, so a study with more VTE events was given greater weight than studies with fewer VTE events. A pooled estimate was also calculated for the third trimester of pregnancy.

Given that diagnosis modalities have improved over the years allowing for better ascertainment of VTE diagnosis in addition to the increasing prevalence of maternal risk factors for VTE (for example obesity), we performed a subgroup analysis by stratifying studies based on calendar year (before and after 2005). This cut-off was based on the initial examination of the forest plot on the incidence of VTE during pregnancy which showed a marked difference in the rate of VTE after 2005. We also stratified our analyses based on whether or not VTE cases were subjected to a degree of validation/confirmation, which varied from study to study, however, we accepted methods similar to our criteria. The methods used to validate/confirm VTE ranged from using a validated algorithm to confirm VTE diagnosis or a registry where VTE cases were previously validated to only including cases where VTE had been objectively confirmed by diagnostic tests. A diagnosis of pulmonary embolism may have been confirmed by pulmonary angiography, CT, Magnetic Resonance Imaging, VQ scan, or pathological confirmation of thrombus. A diagnosis of DVT may have been confirmed by Doppler ultrasound, duplex ultrasonography, venography, or pathological confirmation of thrombus.

The heterogeneity was assessed in terms of *I.* ^2^ All data management and statistical analysis was done using Stata MP11 (Stata Corp., College Station, Texas). This study was approved by the Independent Scientific Advisory Committee (ISAC) that governs use of the CPRD for research (reference number = 10_193R).

## Results

### Cohort analysis

Overall there were 1,117,691 women with follow-up data between the ages of 15 and 44 years experiencing 248,953 pregnancies resulting in live or stillbirths (Table 1). The median follow-up for each women was 3.2 years (IQR = 1.2–6.5).

**Table 1:** Basic characteristics of study population.

Variables	N (%)
Total women in study	1,117,691
Median follow-up (Inter quartile range) in years	3.2 (1.23–6.50)
**Pregnancy outcome (total pregnancies N = 248,953)**	
Live births	247,436 (99.3)
Stillbirths	1,517 (0.61)
**Study follow-up time in person-years (total study period = 4,821,334)**	
Not pregnant	4,613,196
Antepartum	156,541
Time around delivery	2,381
Postpartum	49,216

#### Defining first VTE using linked primary and secondary care data

There were 3,507 cases of first VTE using both data sources. Around 51% of the VTE cases were categorised under VTE definition A of which 51% of diagnoses were recorded both in primary and secondary care data (Table 2). Twenty percent of all VTE cases were categorised under VTE definition B as they had supporting evidence including signs or symptoms or a diagnostic test documented 15 days before or after the date of VTE diagnosis but did not meet our criteria for VTE definition A. A total of 29% of all the VTEs were categorized as VTE definition C, i.e. diagnoses with no supporting evidence, the majority of which were in primary care data. When only using the primary care data to identify first VTE cases, a total of 2,923 cases were identified of which 58%, 19% and 23% where categorised under VTE definition A, B and C respectively (data not shown). Similarly 1,946 potential VTE cases were identified when only using secondary care data to identify first VTE of which 64%, 18% and 18% were categorized as VTE definition A, B and C respectively (data not shown).

**Table 2:** Percentages of VTE cases in the study population according to our three definitions.

Cases of first VTE	N = 3,507 n (%)
**VTE definition A** 1 **Total**	**1,805 (51.0)**
Diagnosis of VTE in HES and CPRD	925 (51.2)*
Diagnosis in HES only	168 (9.4)*
Diagnosis in CPRD only	712 (39.4)*
**VTE definition B** 2 **Total**	**728 (20.6)**
Diagnosis of VTE in HES and CPRD	161 (22.1)*
Diagnosis of VTE in HES only with supporting evidence	162 (22.2)*
Diagnosis of VTE in CPRD only with supporting evidence	405 (55.6)*
**VTE definition C** 3 **Total**	**974 (27.6)**
Only HES with no supporting evidence	316 (32.4)*
Only CPRD with no supporting evidence	658 (67.5)*

1 Recorded VTE with supporting anticoagulant prescription, or medical code indicating anticoagulant therapy within 90 days of the event or death within 30 days of the event.

2 Recorded VTE codes with supporting sign or symptom of VTE within 15 days before or after the date of event.

3 Recorded VTE with no evidence of signs and symptoms or anticoagulant therapy.

* Percentages based on different denominators (in bold).

#### Timing of VTE diagnosis (for cases diagnosed both in primary and secondary care)

Of the total 1,086 VTE cases documented in both primary and secondary care, 35% (n = 377) had the same date of diagnosis for VTE in both datasets. Of the total cases with a different date of diagnosis, 82% (n = 581) were first diagnosed in HES with a median delay of 7 days (IQR = 3–13) until it was recorded in patients' primary care records. For VTE cases first diagnosed in primary care (number of cases = 128) there was a median of 4 days difference (IQR = 1–18) in the recording between primary and secondary care date of VTE. This was broadly the same in the antepartum period, postpartum period and non-pregnant period.

#### Incidence of VTE in and around pregnancy

The rate of any VTE (VTE definition A, B or C) during the time not associated with pregnancy using both primary and secondary care data was 61 per 100,000 person-years (Table 3). This rate decreased by half when restricting to VTE definition A (32 per 100,000 person-years). When relying solely on primary care recording of first VTE, the rate using VTE definition A during antepartum, around delivery and postpartum periods was calculated to be 80, 461 and 324 per 100,000 person-years respectively (Table 4). Relying solely on secondary care data for the recording of first VTE, the calculated VTE rate during the time around delivery and postpartum was calculated to be 1799 and 180 per 100,000 person-years respectively.

**Table 3:** Rate of VTE per 100,000 person-years using different definitions of VTE in and around pregnancy (for pregnancies resulting in live or stillbirths).

Time period	VTE definition A^1^, B^2^ or C^3^		VTE definition A^1^ or B^2^		VTE definition A^1^	
	n	Rate* (95%CI)	n	Rate* (95%CI)	n	Rate* (95%CI)
Not pregnant	2,817	61 (58–63)	2040	44 (42–46)	1480	32 (30–33)
Antepartum	377	240 (217–266)	268	171 (151–192)	156	99 (85–116)
Trimester 1	47	95 (71–127)	41	83 (61–113)	23	46 (31–70)
Trimester 2	76	148 (118–186)	49	95 (74–126)	30	58 (41–83)
Trimester 3	254	450 (398–509)	178	315 (272–365)	103	182 (150–221)
Around delivery	76	3192 (423–546)	38	1596 (1161–2193)	34	1428 (1020–1998)
Postpartum	237	481 (423–546)	187	379 (329–438)	135	274 (231–324)
Early postpartum	200	801 (695–920)	157	629 (537–735)	177	468 (391–561)
Late postpartum	37	151 (109–208)	30	122 (95–175)	18	73 (46–116)

* Rate per 100,000 person-years.

** With women recorded as having a VTE event if they had a diagnosis in either database.

1 Recorded VTE with supporting anticoagulant prescription, or medical code indicating anticoagulant therapy within 90 days of the event or death within 30 days of the event.

2 Recorded VTE codes with supporting sign or symptom of VTE within 15 days before or after the date of event.

3 Recorded VTE with no evidence of signs and symptoms or anticoagulant therapy.

**Table 4:** Incidence rate of VTE per 100,000 person-years in and around pregnancy (for pregnancies resulting in live or stillbirths) by data source.

Time period	VTE definition A^1^	
	n	Rate* (95% CI)
**Using only primary care data to identify first VTE events**		
Not pregnant	1387	30 (28–31)
Antepartum	126	80 (67–95)
Trimester 1	22	44 (29–68)
Trimester 2	31	60 (42–86)
Trimester 3	73	129 (102–162)
Around delivery	11	461 (255–833)
Postpartum	160	324 (278–379)
Early postpartum	138	552 (467–652)
Late postpartum	22	898 (591–136)
**Using only secondary care data to identify first VTE events**		
Not pregnant	985	21 (19–22)
Antepartum	123	78 (65–93)
Trimester 1	18	36 (23–58)
Trimester 2	25	48 (32–72)
Trimester 3	80	141 (113–176)
Around delivery	43	1799 (1334–2426)
Postpartum	89	180 (146–221)
Early postpartum	76	303 (242–279)
Late postpartum	13	52 (30–91)

* Rate per 100,000 person-years.

**Figure 1:**
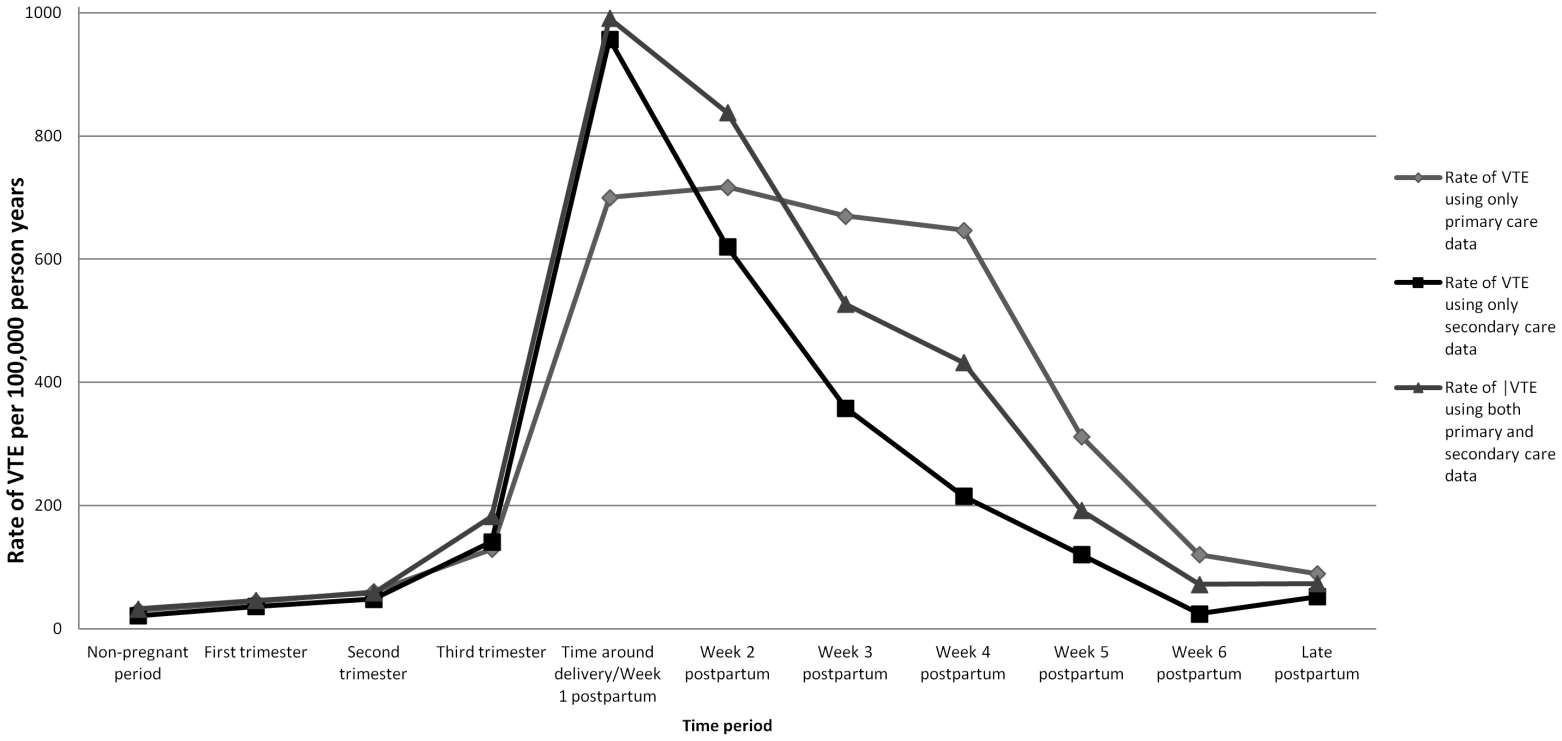
Rate of VTE in and around pregnancy and and non-pregnant periods using only VTE definition A.

During the early postpartum period, the observed rate of VTE using both primary and secondary care data, peaked around the time of delivery and the first week of postpartum period (AR = 991 per 100,000 person years) after which the rates showed a graded decline throughout the remaining postpartum period (Figure 1). Compared to rates from the combined data sources, secondary care data showed a similar rate around delivery but much lower rates postpartum that decreased more rapidly following delivery. In contrast, primary care data showed lower rates around delivery but higher postpartum rates that remained consistently high until 4 weeks postpartum.

### Systematic review and meta-analysis of perinatal VTE incidence studies

**Figure 2:**
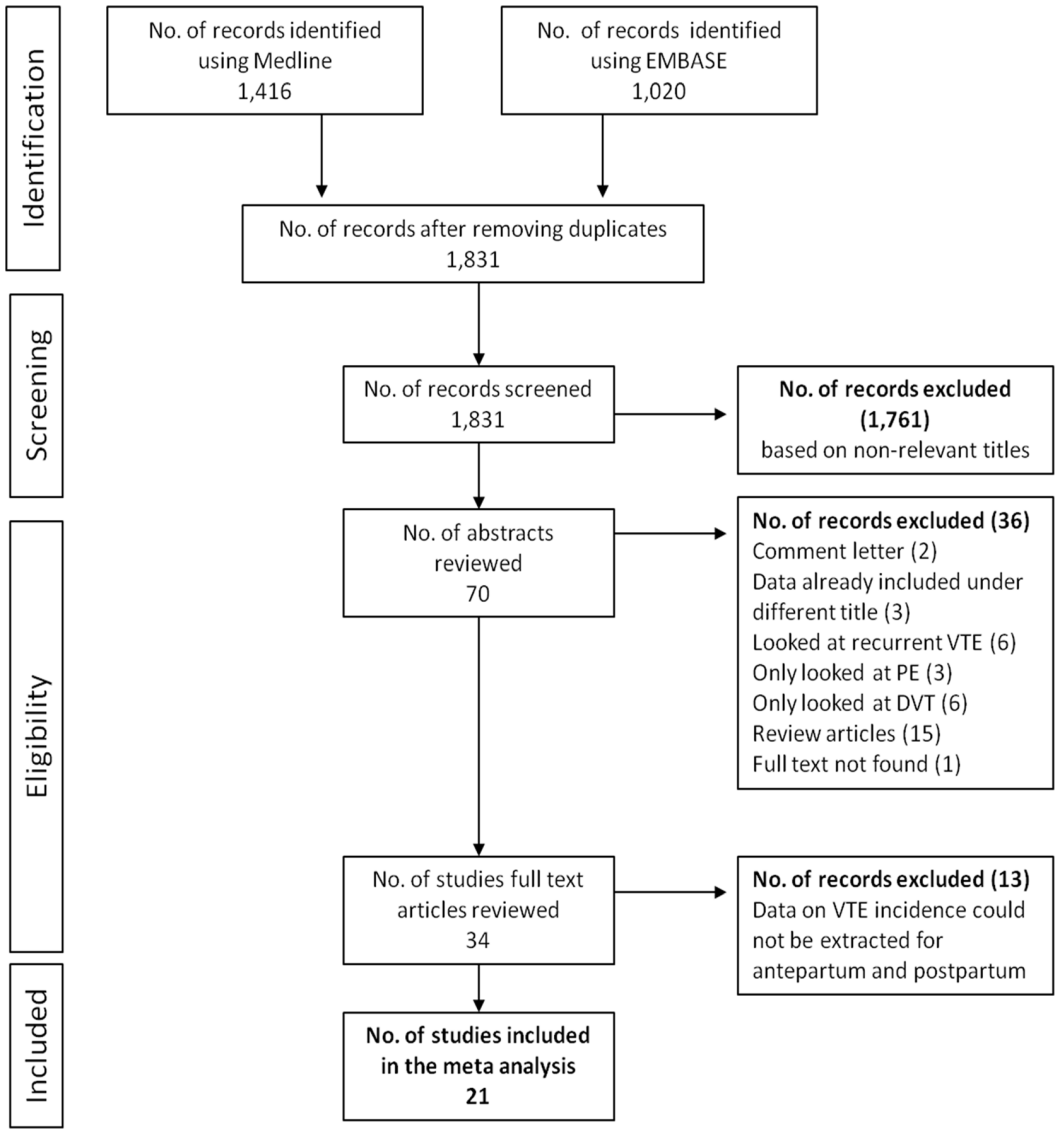
PRISMA flow diagram for identification of VTE incidence studies during pregnancy, postpartum or both.

**Figure 3:**
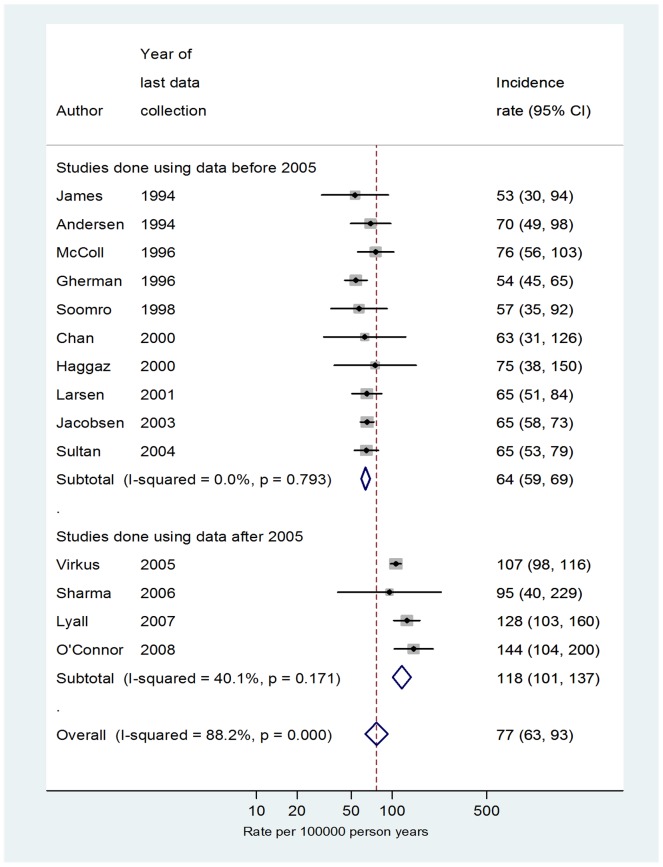
Rate of VTE per 100,000 person years during the antepartum period only including studies where some degree of case validation/confirmation was used. The data were stratified by year.

**Figure 4:**
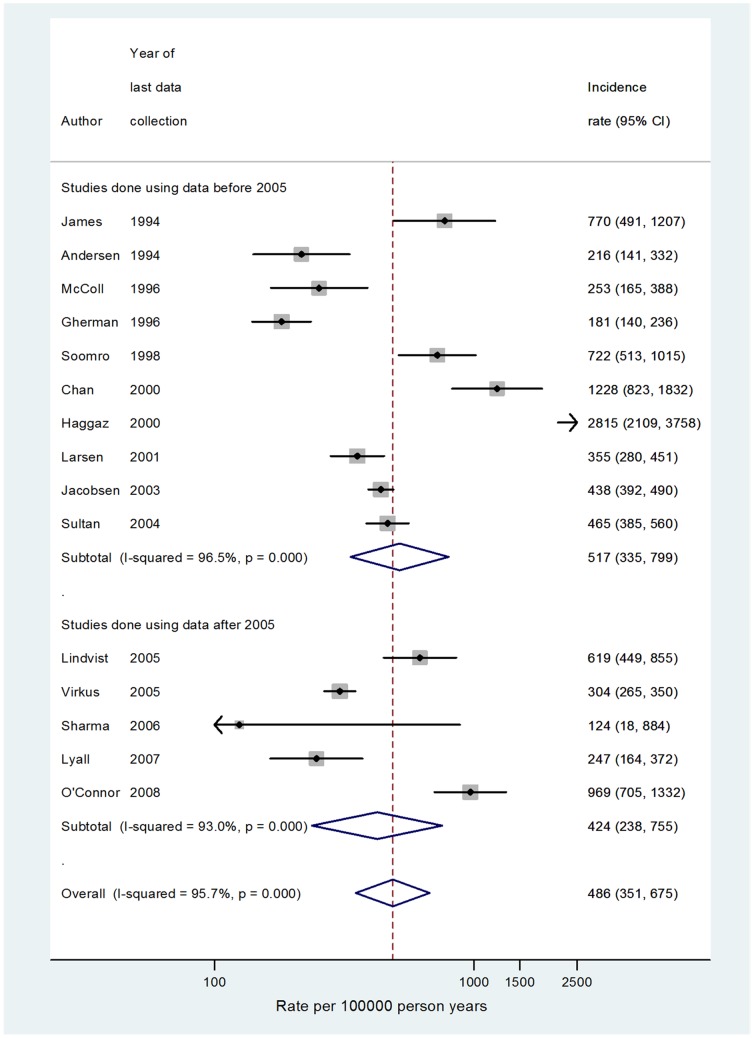
Rate of VTE per 100,000 person years during the postpartum (first six week after childbirth) period only including studies where some degree of case validation/confirmation was used. The data were stratified by year.

We identified 1,831 articles of which 34 had their full-texts reviewed and 21 were eventually included (Figure 2). The characteristics of the included studies are presented in Table 5. The incidence rate of VTE during the antepartum period from previous studies ranged from 37 per 100,000 person-years in the UK to 144 per 100,000 person-years in the U.S.A (data not shown). The pooled incidence rate of VTE during the antepartum period was 76 per 100,000 person-years (95% CI 65–90; heterogeneity *I* ^2^ = 97.6%). When restricting to studies where VTE cases were validated/confirmed (14 studies) we found a higher incidence of VTE after 2005 (AR = 118 per 100,000 person-years *I* ^2^ = 40%) compared to the rate before 2005 (AR = 64 per 100,000; *I* ^2^ = 0.0%) for the antepartum period. (Figure 3). The pooled absolute rate of VTE during the third trimester of pregnancy post 2005 (based on 389 VTE events; data not shown) was calculated to be 142 per 100,000 person-years (95% CI 93–158; *I* ^2^ = 70%) when a similar restriction was applied. Similarly the pooled absolute rate of VTE during the first six weeks postpartum (post 2005) was calculated to be 424 per 100,000 person-years (95%CI 238–755; *I* ^2^ = 96%; Figure 4).

**Table 5:** Characteristics of the studies included by year of publication.

Author (Year of publication)	Country	Study period	Study design and methodology	Measures taken to confirm VTE
Sultan [14] (2012)	United Kingdom	1987–2004	Population based retrospective cohort. Cases were identified using primary care data which were validated	VTE cases were confirmed based on anticoagulant therapy
Vikrus [19] (2011)	Denmark	1995–2005	Population based retrospective cohort. Cases were identified from national registry using ICD-10 codes.	Registry was validated for VTE during pregnancy with PPV of more than 80%
O'Cornnor [29] (2011)	United States	2003–2008	Cross sectional study. Cases were identified from a single hospital.	VTE cases were objectively confirmed based on diagnostics tests^1^
Liu [30] (2009)	Canada	1991–2006	Cross sectional study. Cases were identified using hospital discharge database using ICD-10 codes.	No measures taken to confirm VTE
Lyall [31] (2008)	United Kingdom	1999–2007	Cross sectional study. Cases were identified from a single unit	VTE cases were objectively confirmed based on diagnostics tests^1^
Jacobsen [25] (2008)	Norway	1990–2003	Cross sectional study. Cases were identified from patient and birth register.	VTE cases were validated for the subset of the data
Lindqvist [32] (2008)	Sweden	1990–2005	Cross sectional study. Cases were identified from a single hospital.	VTE cases were objectively confirmed based on diagnostics tests^1^
Sharma [27] (2008)	Australia	1999–2006	Cross sectional study. Cases were identified from a single hospital.	VTE cases were objectively confirmed based on diagnostics tests^1^
Larsen [33] (2007)	Denmark	1980–2001	Cross sectional study. Cases were identified from a single county.	VTE cases were objectively confirmed based on diagnostics tests^1^
James [20] (2006)	United States	2000–2001	Cross-sectional study. Cases were identified from national inpatient sample which covers around 100 hospitals using ICD-10 codes.	VTE cases were objectively confirmed^1^. Included some probable and possible VTEs
Heit [34] (2005)	United States	1966–1995	Population based cohort. Potentially fertile women were prospectively followed in a single county.	VTE cases were objectively confirmed^1^. Included some probable and possible VTEs
Haggaz [35] (2003)	Sudan	1999–2000	Cross sectional study. Cases were identified from a single hospital.	VTE cases were objectively confirmed based on diagnostics tests^1^
Anderson [36] (2003)	Denmark	1984–1994	Cross sectional study. Cases were identified using inpatient registry.	VTE cases were objectively confirmed based on diagnostics tests^1^
Soomro [37] (2002)	Saudi Arabia	1986–1998	Cross sectional study. Cases were identified from a single hospital.	VTE cases were objectively confirmed based on diagnostics tests^1^
Chan [24] (2001)	Japan	1998–2000	Cross sectional study. Cases were identified from a single hospital.	VTE cases were objectively confirmed based on diagnostics tests^1^
Ros [1] (2001)	Sweden	1987–1995	Population based retrospective cohort. Cases were identified using inpatient registry using ICD-9 codes	No measures taken to confirm VTE
Simpon [21] (2001)	United Kingdom	1988–1997	Cross sectional study in which cases were identified using ICD codes from hospital database	No measures taken to confirm VTE
Gherman [26] (1999)	United States	1978–1996	Cross sectional study. Cases were identified from a single hospital.	VTE cases were objectively confirmed based on diagnostics tests^1^
Lindqvist [38] (1999)	Sweden	1990–1993	Cross sectional study. Cases were identified from Swedish birth and patient registry	No measures taken to confirm VTE
McColl [39] (1997)	United Kingdom	1985–1996	Cross sectional study. Cases were identified using national health service data.	VTE cases were objectively confirmed based on diagnostics tests^1^
James [40] (1996)	United States	1989–1994	Cross sectional study where cases were identified from medical records at a single hospital.	No measures taken to confirm VTE

1 A diagnosis of pulmonary embolism may be confirmed by pulmonary angiography, CT, MRI, ventilation-perfusion scan, pathological confirmation of thrombus etc. whereas the diagnosis of DVT may be confirmed by Doppler ultrasound, duplex ultrasconography, venography, pathological confirmation of thrombus etc. each of which may vary from study to study.

### Comparison of meta-analysis estimates to the cohort analysis

The estimates from the meta-analysis post-2005 are than lower those generated using the inclusive VTE definition (VTE definition A, B or C and VTE definition A or B). However using only cases categorised under VTE definition A (those confirmed based on prescriptions or death), the incidence rate of 99, 182 and 468 during the antepartum period, third trimester of antepartum and early postpartum period (first six weeks after birth) from our cohort analysis are in concordance with the pooled estimates from recent studies (118, 142 and 424 respectively).

## Discussion

### Main findings

In this study, we have shown that a highly specific definition of VTE (VTE definition A) using data from both primary and secondary care health care settings is required to accurately estimate the incidence of VTE in and around pregnancy. The estimates we have derived are comparable with the pooled incidence rates for antepartum and early postpartum periods from the existing literature. With the use of linked primary and secondary care data we were able to get a far more accurate date of diagnosis for VTE which resulted in more precise estimates of rates close to delivery. Whilst the VTE rate can be accurately estimated in the separate data sources in some circumstance (e.g. trimesters of antepartum), when relying solely on primary care data we found that the rate of VTE was much lower during the time around delivery but higher during the postpartum period compared to solely using secondary care data. This difference is likely because of a delay in hospitalised events being recorded in primary care. However when solely relying on secondary care data the rate of VTE was much lower during the postpartum and non-pregnant periods. We believe the best method for defining and quantifying the incidence of VTE in and around pregnancy is therefore to use both primary and secondary care data and apply our VTE definition A to identify cases, as it has previously been validated [3] and is externally comparable to other pregnancy studies.

### Ascertainment of VTE events

In our study, we were able to analyse 1,117,689 women of childbearing age and over 248,000 pregnancies to determine how the incidence of VTE in and around pregnancy varies based on the VTE definition and the dataset used; such analysis has not been done before. Our VTE definition A mimics a previously validated VTE definition in primary care CPRD [3] data in a non-pregnant population with a reported positive predictive value of 84%. To this algorithm we added secondary care diagnosis data which has an overall accuracy of 91% [17]. One current drawback of UK secondary care data is the lack of information on hospital prescribed heparin and warfarin which may have lead to under ascertainment of cases using VTE definition A. However, we believe that the impact of this limitation should be minimal as pregnant women with a VTE diagnosis are expected to be on anticoagulation therapy throughout their pregnancies and this therapy period extends up to three months for postpartum women [18]. Therefore these prescriptions are likely to be captured in the primary care data. We must acknowledge that 33% of pregnancies had no information on the length on gestation. When we conducted a sensitivity analysis, however, and calculated the rate of VTE stratified by those with and without information on gestational age this showed no difference in our estimates of VTE within each trimester of pregnancy.

### Date of VTE diagnosis

Another strength of linked data is the improvement in estimation of the date of diagnosis of VTE. The majority (84%) of the VTEs diagnosed in both primary and secondary care using the linked data had the diagnosis made in secondary care data first. Therefore if we were reliant on primary care data alone there would be a concern with the delay in recording of VTE from secondary to primary care. Our study demonstrated a median lag of 7 days in the recording of VTE events from secondary to primary care. This delay can restrict the ability to give precise incidence estimates in narrow windows of time such as around delivery and probably explains our low incidence rate during the time around delivery and prolonged high risk during the early weeks of postpartum when only relying on primary care data for VTE diagnosis. One potential limitation of the secondary care data is the reliance on episode start date as the date of VTE event. This creates problems in separating out antepartum versus postpartum VTE events around the time of delivery. An example of this problem is that reported in a cohort study of women delivering in hospital by Virkus et.al [19] who considered the date of admission to hospital as the date of diagnosis for VTE. They reported a high rate of VTE during the third trimester of the antepartum compared to previous studies in the meta-analysis post 2005. This may be explained by some postpartum VTE events occurring during the maternal admission having been classified as antepartum. A similar, yet not acknowledged, problem may be the case for other studies utilizing hospital discharge data [20], [21]. In our study, HES provides a better option in terms of date of episodes for each diagnosis within each hospital period. We think this rather than the date of hospital admission or discharge will more accurately estimate the true VTE diagnosis date (although not necessarily the actual biological onset of the VTE). Furthermore the division of pregnancy periods as antepartum, around delivery, and postpartum adequately addresses the concern of mis-classification of VTE events around childbirth.

### Meta-analysis

Our meta-analyses showed high levels of heterogeneity occurring among the individual studies for both antepartum and postpartum pooled estimates. In descriptive epidemiological studies where a statistic is estimated among a single group (such as pregnant women in this review), the potential for heterogeneity is far greater than for analytic or comparative studies (i.e., when two groups are compared to calculate a measure of effect such as an odds or risk ratio). This is because incidence rates are very sensitive to the choice of study population, outcome definition and dataset used meaning at least some heterogeneity will be inevitable; other published meta-analyses of this type also report very high levels of heterogeneity [15], [22], [23]. The heterogeneity in our data during the antepartum period was partially explained by calendar year and whether VTE cases were subjected to a certain degree of validation/confirmation or not. For instance, when we restricted our analysis only to studies where VTE cases were validated/confirmed and stratified them by calendar year, our *I^2^* value was less than 50%. For the postpartum, incidence rates during the first six weeks post-delivery were largely inconsistent even after restricting to studies with validated/confirmed VTE and stratifying the estimates by calendar year. This wide variation in the reported rates can probably be explained by the various countries' health care systems and their thromboprophylaxis practices after childbirth. The UK Royal College of Obstetricians and Gynaecologists recommendation on VTE risk assessment and thromboprophylaxis post-caesarean section dates back to 1993, which may have been adapted by different countries at different points in time. For instance a study from China [24] reported the rate of VTE to be 1228 per 100,000 person-years where there was no concept of thromboprophylaxis prior to the year 2000 versus studies from Norway [25] and UK [14] with lower reported rates (AR around 400 per 100,000 person-years). This is something we were not able to account of in our meta-analysis for postpartum VTE. Most of the previous literature on this subject has relied on secondary care data which will inevitably miss many non-fatal VTE events diagnosed and managed exclusively in primary care, particularly during the postpartum period [1], [19], [26], [27].

Although we did no external validation of our VTE definitions among women included in our cohort study, based on our most inclusive definition (i.e. including events categorised under VTE definition A, B or C) our calculated rates of VTE during antepartum and postpartum periods were considerably higher than the pooled incidence rate of previous studies. This suggests the inclusion of many false positive events, limiting the validity of such an inclusive VTE definition. The same issue occurred, although to a lesser extent, when including VTE events classified under VTE definition A or B where we included cases with clinical signs and symptoms of VTE or evidence of diagnostic tests in addition to anticoagulant therapy. This may be due to the fact that leg swelling and calf pain are common in the third trimester of pregnancy in women without DVT which can lead to potential misclassification. Additionally, D-dimer levels increase [28] in pregnancy, with gestational hypertension, and in preterm labour leading to false positive events which may add to that misclassification. In contrast the absolute rates of VTE using VTE definition A for antepartum and postpartum periods of 99 and 468 per 100,000 person-years respectively are broadly in concordance with pooled estimates from previous studies using similar methodology where VTE cases were validated/confirmed.

## Conclusions

Our results have important implications for the way in which VTE is studied in pregnancy using routinely available electronic health care records, data which are crucial for assessing outcomes that are severe and rare and thus rely on evidence from large population-based sources. Firstly, we have quantified the incidence of VTE in and around pregnancy using a variety of VTE definitions. This demonstrated that the absolute rate of VTE greatly varies based on the VTE definition used, with our VTE definition A providing the most comparable estimates of the absolute rates to previous work. We have also demonstrated that there are some important limitations in using solely primary care or secondary care data in terms of the date and ascertainment of VTE diagnosis which need to be considered when interpreting studies that do this. We have shown in our study that using both primary and secondary care data not only provide better estimation of the date of VTE diagnosis, but also enable researchers to comprehensively identify VTE cases diagnosed and recorded both in primary or secondary care. Furthermore, the use of both primary and secondary care data combined may provide better ascertainment of maternal risk factors for VTE, information on hospitalization, primary care prescriptions, information on life style related factors and co-morbidities. This vital information could be used to better understand the occurrence and risk factors of VTE in and around pregnancy for future research

## Supporting Information

Table S1 **Search strategy used for Medline database.**
(DOCX)

Table S2 **Search strategy used for Embase database.**
(DOCX)
