# Supplementary material for: The Incidence of First Venous Thromboembolism in and around Pregnancy Using Linked Primary and Secondary Care Data: A Population Based Cohort Study from England and Comparative Meta-Analysis
Source: PLoS One. 2013 Jul 29;8(7):e70310. doi: 10.1371/journal.pone.0070310 (PMC3726432; doi:10.1371/journal.pone.0070310)
Supplement: Table S1 — Search strategy used for Medline database. (DOCX) [file pone.0070310.s001.docx]

**Table S1: Search strategy used for Medline database**

| **No.** | **Terms** |
| --- | --- |
| 1 | epidemiologic studies.mp. [mp=title, abstract, original title, name of substance word, subject heading word, keyword heading word, protocol supplementary concept, rare disease supplementary concept, unique identifier] |
| 2 | exp cohort studies/ |
| 3 | (cohort adj (study or studies)).tw. |
| 4 | Epidemiologic Studies/ |
| 5 | Cohort Studies/ |
| 6 | (follow up adj (study or studies)).tw. |
| 7 | (observational adj (study or studies)).tw. |
| 8 | logitudinal.tw. |
| 9 | retrospective.tw. |
| 10 | Incidence/ |
| 11 | exp Case-control studies/ |
| 12 | case control.tw. |
| 13 | or/1-12 |
| 14 | exp Venous thromboembolism/ |
| 15 | exp Venous Thrombosis/ |
| 16 | exp Thrombosis/ |
| 17 | exp Pulmonary Embolism/ |
| 18 | exp deep vein thrombosis/ |
| 19 | (dvt$ or (deep adj8 (vein$ or ven$) adj8 thromb$) or embol$).mp. [mp=title, abstract, original title, name of substance word, subject heading word, keyword heading word, protocol supplementary concept, rare disease supplementary concept, unique identifier] |
| 20 | 14 or 15 or 16 or 17 or 18 or 19 |
| 21 | exp pregnancy/ |
| 22 | exp antepartum/ |
| 23 | exp postpartum/ |
| 24 | 21 or 22 or 23 |
| 25 | 13 and 20 and 24 |
| 26 | limit 25 to yr="1960 -Current" |
| 27 | limit 26 to english language |
| 28 | limit 27 to humans |
