# Supplementary material for: The Incidence of First Venous Thromboembolism in and around Pregnancy Using Linked Primary and Secondary Care Data: A Population Based Cohort Study from England and Comparative Meta-Analysis
Source: PLoS One. 2013 Jul 29;8(7):e70310. doi: 10.1371/journal.pone.0070310 (PMC3726432; doi:10.1371/journal.pone.0070310)
Supplement: Table S2 — Search strategy used for Embase database. (DOCX) [file pone.0070310.s002.docx]

**Table S2: Search strategy used for Embase database**

| **No.** | **Terms** |
| --- | --- |
| 1 | clinical study/ |
| 2 | case control study/ |
| 3 | family study/ |
| 4 | longitudinal study/ |
| 5 | retrospective study/ |
| 6 | Prospective study/ |
| 7 | Randomized controlled trials/ |
| 8 | 6 not 7 |
| 9 | Cohort analysis/ |
| 10 | (Cohort adj (study or studies)).mp. |
| 11 | (Case control adj (study or studies)).tw. |
| 12 | (follow up adj (study or studies)).tw. |
| 13 | (observational adj (study or studies)).tw. |
| 14 | (epidemiologic$ adj (study or studies)).tw. |
| 15 | (cross sectional adj (study or studies)).tw. |
| 16 | or/1-5,8-15 |
| 17 | exp pregnancy/ |
| 18 | antepartum.mp. |
| 19 | antenatal.mp. |
| 20 | postpartum.mp. |
| 21 | postnatal.mp. |
| 22 | 17 or 18 or 19 or 20 or 21 |
| 23 | (dvt$ or (deep adj8 (vein$ or ven$) adj8 thromb$) or embol$).mp. [mp=title, abstract, subject headings, heading word, drug trade name, original title, device manufacturer, drug manufacturer, device trade name, keyword] |
| 24 | exp embolism/ or exp thromboembolism/ or exp thrombosis/ or exp venous thromboembolism/ or exp vein thrombosis/ or exp lung embolism/ |
| 25 | exp pulmonary embolism/ |
| 26 | exp deep vein thrombosis/ |
| 27 | 23 or 24 or 25 or 26 |
| 28 | 16 and 22 and 27 |
| 29 | limit 28 to english language |
| 30 | limit 29 to human |
